# *Bartonella quintana* Endocarditis in Persons Experiencing Homelessness, New York, New York, USA, 2020–2023

**DOI:** 10.3201/eid3012.240433

**Published:** 2024-12

**Authors:** Marina Keller, Mariam Agladze, Tania Kupferman, Shannan N. Rich, Grace E. Marx, Rachel Gnanaprakasam, Rich Kodama, Marta Feldmesser, Kara Mitchell, Danielle Wroblewski, Stefan Juretschko, George M. Kleinman, Matthew J. Kuehnert, Julu Bhatnagar, Marlene Deleon Carnes, Hannah Bullock, Sarah Reagan-Steiner, Gabriella Corvese, Joel Ackelsberg

**Affiliations:** Westchester Medical Center, Valhalla, New York, USA (M. Keller, R. Gnanaprakasam, G.M. Kleinman); New York University Grossman School of Medicine, New York, New York, USA (T. Kupferman, M. Agladze); Centers for Disease Control and Prevention, Fort Collins, Colorado, USA (S.N. Rich, G.E. Marx); Memorial Sloan Kettering Cancer Center, New York (R. Kodama); Lenox Hill Hospital, New York (M. Feldmesser); New York State Department of Health, Albany, New York, USA (K. Mitchell, D. Wroblewski); Northwell Health Laboratories, Little Neck, New York, USA (S. Juretschko); Centers for Disease Control and Prevention, Atlanta, Georgia, USA (M.J. Kuehnert, J. Bhatnagar, M.D. Carnes, H. Bullock, S. Reagan-Steiner); New York City Department of Homeless Services, New York (G. Corvese); New York City Department of Health and Mental Hygiene, Long Island City, New York. USA (J. Ackelsberg)

**Keywords:** Bartonella quintana, Bartonella, endocarditis, homelessness, bacteria, bacterial infection, vector-borne, zoonosis, New York, United States

## Abstract

*Bartonella quintana* infection can lead to bacillary angiomatosis, peliosis hepatis, chronic bacteremia, and culture-negative endocarditis. Transmitted by the human body louse (*Pediculus humanus humanus*), *B. quintana* infection has become an emerging disease in recent decades among persons experiencing homelessness. By using retrospective laboratory surveillance, we identified 5 cases of left-sided, culture-negative *B. quintana* endocarditis among persons in New York, New York, USA, during January 1, 2020–November 23, 2023. Identifications were made by using molecular assays. All patients experienced unsheltered homelessness in the year before hospitalization. Of those patients, 4 experienced heart failure, 3 renal failure, and 2 embolic strokes; 2 died. Aortic valve replacement occurred in 4 cases. A history of possible body louse infestation was found in 4 cases. Clinicians should consider housing status and history of lice exposure in patients with suspected bartonellosis and have a low threshold for diagnostic testing and empiric treatment in patients experiencing homelessness.

*Bartonella* bacteria are fastidious, intracellular gram-negative rods that cause disease in humans, cats, dogs, rodents, and other mammals. Thirteen *Bartonella* species are known to cause human infection (*1*). *B. quintana* infection can cause bacillary angiomatosis, peliosis hepatis, chronic bacteremia (*2*–*4*), and culture-negative endocarditis (*5*,*6*). *B. quintana* is transmitted by the human body louse (*Pediculus humanus humanus*) through inoculation of broken skin by contaminated feces. Infection is enabled in settings where personal hygiene is compromised, such as refugee camps (*7*) and wartime environments (*8*). In recent decades, *B. quintana* infection has emerged as a disease primarily affecting persons experiencing homelessness (*3*,*5*,*9*–*20*).

In January 2023, *B. quintana* infection was diagnosed in 2 kidney transplant recipients from 1 donor (*21*). An investigation by the New York City (NYC) Department of Health and Mental Hygiene (New York, NY, USA) determined the donor had experienced unsheltered homelessness and had *B. quintana* bacteremia during their terminal hospitalization in summer 2022. In April 2023, another case of *Bartonella* endocarditis diagnosed in a patient experiencing homelessness was reported to the NYC Department of Health and Mental Hygiene, prompting efforts to identify additional cases to better understand the incidence and severity of *B. quintana* infections in the city (*22*). This article provides information regarding the clinical manifestations, diagnosis, and treatment of each case and discusses management options for clinicians caring for patients experiencing homelessness who might have bartonellosis.

## Methods

The NYC Department of Health and Mental Hygiene conducted retrospective active surveillance for patients with a diagnosed *B. quintana* infection among clinical laboratories of 5 large hospital networks and the Wadsworth Center at the New York State Department of Health. The laboratories identified patients during January 1, 2020–November 23, 2023, who had a *Bartonella* spp. recovered from specimen cultures; who tested positive for *Bartonella* spp. with PCR further confirmed as *B. quintana* by 16S rRNA sequencing; whose plasma yielded >10 molecules/μL of *B. quintana* cell-free DNA detected with next-generation sequencing (*23*); or whose serum demonstrated *B. quintana* or *B. henselae* IgM or IgG titer results of >1:128. All serologic testing was conducted at the same commercial clinical laboratory.

We reviewed the electronic medical records of patients identified by the laboratories, accessed by using NYC regional health information organizations for clinical and social history details, which included documentation of housing status. The NYC Department of Homeless Services registration database was queried for evidence that cases received services in the NYC shelter system. The NYC Department of Health and Mental Hygiene’s Institutional Review Board determined that the activities described in this article meet the definition of public health surveillance as set forth under 45 CFR§46.102(1)(2). 

## Results

We identified 5 cases of culture-negative, left-sided *B. quintana* endocarditis. The 5 patients had experienced unsheltered homelessness within 1 year of admission. None were registered in the NYC shelter system. The case descriptions illustrate similarities in clinical manifestations and highlight the concern of delayed diagnosis.

### Case 1 Description

In summer 2022, a middle-aged man with schizophrenia and alcohol use disorder was found unresponsive at an assisted living facility where he had lived for ≈1 year for the management of malnutrition and chronic anemia. According to his family, the patient had previously been living unsheltered in NYC and had been treated for body lice. In the emergency department, the patient was unresponsive but had unremarkable vital signs (Table). Laboratory testing revealed severe anemia and renal failure. Computed tomography (CT) scans of the brain revealed a large, acute, right inferior cerebellar intraparenchymal hemorrhage with surrounding edema. Cerebral angiography revealed a 3-mm right posterior inferior cerebellar artery aneurysm that was treated emergently with endovascular coiling.

**Table:** Demographic and clinical features of 5 *Bartonella quintana* endocarditis cases in persons experiencing homelessness, New York, New York, USA, 2020–2023*

Characteristics	Case 1	Case 2	Case 3	Case 4	Case 5
Patient demographics					
Age, y	≈50	≈50	≈70	≈50	≈60
Sex	M	M	F	M	M
Race/ethnicity	Black	Middle Eastern	Unknown	Hispanic	Asian
Admission values					
Temperature at admission, °C	37.0	36.2	36.7	36.4	36.9
Heart rate, beats/min	118	101	46	92	72
Respiratory rate, breaths/min	20	19	9	16	20
Blood pressure, mm Hg	138/108	123/51	82/45	111/66	138/85
Leukocytes, 10^3^ cells/mL	8.6	11.1	17	3.1	9.5
Hemoglobin g/dL	6.2	10.3	5.8	8.6	7.8
Platelets, 10^3^/mL	233	205	155	123	350
Creatinine, mg/dL	2.8	1.9	1.4	1.3	0.8
Albumin, g/dL	2.9	2.5	2.5	2.7	3.4
Pro-BNP, pg/mL	Not tested	Not tested	2756	40510	Not tested
Diagnoses and course of illness					
Heart failure	Yes	Yes	Yes	Yes	No
Affected heart valve(s)	Aortic, mitral	Aortic	Aortic	Aortic	Aortic, mitral
Valve replacement	Yes	Yes	Yes	Yes	No
Renal failure	Yes	Yes	Yes	No	No
Renal replacement therapy	No	Yes	Yes	No	No
* B. quintana* IgG titer	>1:1,024	1:1,280	<1:128	1:640	1:2,560
* B. henselae* IgG titer	>1:1,024	1:2,560	>1:1,024	1:2,560	1:2,560
* B. quintana* IgM titer	Negative	Negative	Negative	Negative	Negative
* B. henselae* IgM titer	Negative	Negative	Negative	Negative	Negative
Molecular diagnostic assay	HN-PCR valve	PCR/16S rRNA valve	PCR/16S rRNA valve	16S rRNA gene sequencing	cfDNA, 14049 MPM
Molecular diagnosis	*B. quintana*	*B. quintana*	*B. quintana*	*B. quintana*	*B. quintana*
Antimicrobial drugs administered before *B. quintana* treatment started	VAN, CTX	DAP, CTX	VAN, P/T, CTX	VAN, CTX	VAN, CTX
Days before *B. quintana* treatment started	40	4	3	3	8
Hospitalization, d	46	79	34	33	31
Died during hospitalization	No	No	Yes	No	No
Died within 1 year	Yes	No	Yes	No	No
Risk factors					
Alcohol use disorder	Yes	Yes	Unknown	Yes	No
Illicit drug use	No	Yes	Unknown	Yes	No
Schizophrenia	Yes	No	Unknown	No	No
Bipolar disorder	No	Yes	Unknown	Yes	No
Other mental health condition	Unknown	Yes	Unknown	Unknown	No
Unsheltered homelessness at admission	No	Yes	Presumed	Yes	Yes
Unsheltered homelessness within 1 year of admission	Yes	Yes	Presumed	Yes	Yes
Known exposure to body lice	Yes	Yes	bed bugs	Yes	No
*cfDNA, microbial cell-free DNA detection with next-generation sequencing; CTX, ceftriaxone; DAP, daptomycin; HN-PCR, hemi-nested PCR confirmed with sequence analysis; MPM, DNA molecules per microliter (number of DNA fragments present in 1 microliter of plasma); PCR/16S rRNA, real-time PCR of *gltaA* confirmed with full gene sequencing of PCR product; pro-BNP, pro-B-type natriuretic peptide; P/T, piperacillin/tazobactam.					

Transthoracic echocardiogram (TTE) revealed a 2.2 cm vegetation on the noncoronary aortic valve (AV) leaflet and a 2.0 cm vegetation on the anterior mitral valve leaflet, which is associated with severe regurgitation of both valves with preserved left ventricular function and suggestive of bacterial endocarditis. Healthcare providers collected 1 set of blood cultures before antimicrobial administration and 4 sets the first week of hospitalization. Bacterial cultures were held for 5 days, and fungal cultures were held for 30 days. Cultures remained negative. Providers treated the patient empirically for bacterial endocarditis with ceftriaxone and vancomycin for 12 days, then stopped after blood cultures remained negative. Providers did not initially consider bartonellosis.

The patient received anticoagulants and underwent aortic and mitral bioprosthetic valve replacements 38 days after admission and after neurologic recovery. After reassessment of the case by a new consultant 2 days after surgery, doxycycline was started empirically for possible bartonellosis, and serologic testing was conducted. Results of bacterial culture of the explanted valve tissue was negative; fungal and mycobacterial cultures were not performed. *B. henselae* and *B. quintana* IgG titers were >1:1024; IgM titers were negative.

The patient was discharged and then readmitted 3 weeks later with renal failure. Doxycycline monotherapy was continued for 4 weeks and then switched to azithromycin for an additional 2 weeks because of a drug rash while on doxycycline.

**Figure 1:**
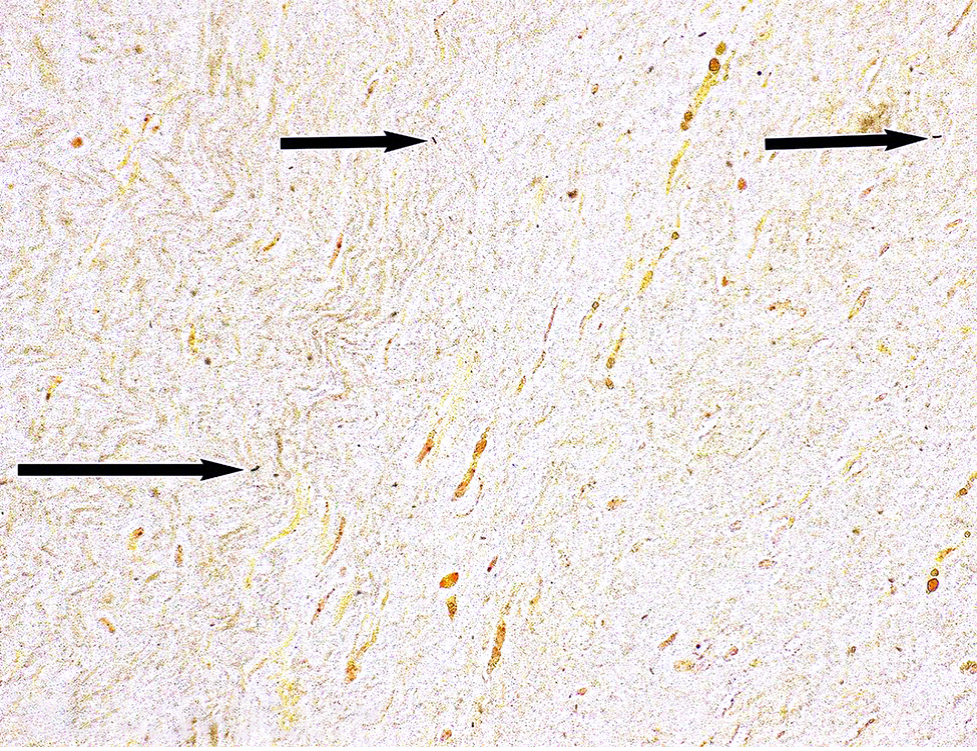
Warthin-Starry staining of aortic valve vegetation from a person experiencing homelessness who had *Bartonella quintana* endocarditis, New York, New York, USA. The presence of argyrophilic microorganisms is consistent with *Bartonella* (arrow). Original magnification ×500.

After the second hospital discharge, a nonspecific qualitative PCR at a commercial laboratory detected a *Bartonella* sp. in an explanted AV tissue specimen. The Wadsworth Center conducted *Bartonella* PCR of whole blood that was collected before doxycycline was started, and the PCR was negative. A Warthin-Starry silver stain of the AV tissue revealed small, black, curved organisms that were consistent with *Bartonella* spp. (Figure 1).

The patient’s family was contacted after he missed outpatient appointments. They reported that he had left home and was experiencing homelessness. The patient sought treatment at another hospital 2 months after the second discharge. The hospital clinicians diagnosed *Clostridioides difficile*–associated diarrhea and *Enterococcus faecalis* bacteremia. No additional *Bartonella* testing was conducted. The patient was discharged to hospice and died 10 months after his endocarditis diagnosis.

**Figure 2:**
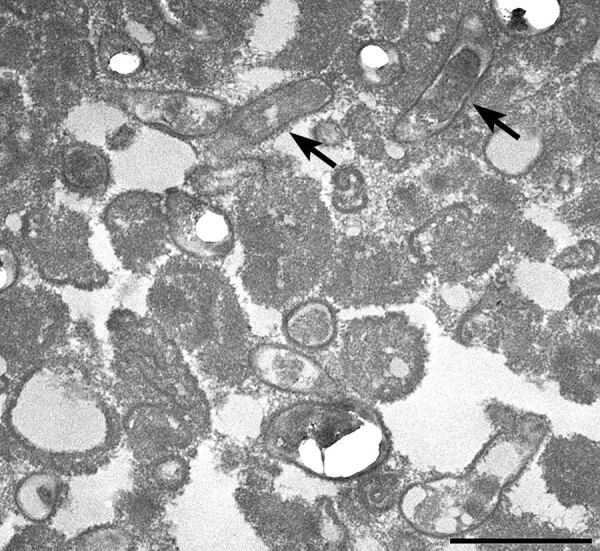
Transmission electron microscopy image from a person experiencing homelessness who had *Bartonella quintana* endocarditis, New York, New York, USA. Image reveals pleomorphic bacteria from a formalin-fixed paraffin-embedded tissue section. Arrows indicate 2 rod-shaped bacteria present in the aorta of the patient. Scale bar indicates 2 μm.

Several months after the patient’s death, formalin-fixed paraffin-embedded explanted AV tissue was submitted to the Centers for Disease Control and Prevention for testing. *B*. *quintana* was identified by using a *Bartonella*-specific hemi-nested PCR targeting the *ribC* gene (*24*) followed by sequence analysis of 307 bp and 211 bp PCR products. Electron microscopy revealed *B. quintana* within aortic tissue (Figure 2).

### Case 2 Description

In early spring 2022, a middle-aged man with chronic obstructive pulmonary disease who had been experiencing unsheltered homelessness sought care at an emergency department with dyspnea on exertion that progressed to shortness of breath at rest over the previous 2 weeks. He had a history of bipolar disorder, antisocial personality disorder, and alcohol use disorder. Body lice were discovered and treated after admission.

Physical examination revealed bilateral lower extremity and scrotal edema and diffuse pulmonary crackles. The patient was tachycardic (Table). Laboratory testing revealed hematuria, mild neutrophilic leukocytosis, acute kidney injury, and metabolic acidosis. A chest radiograph showed pulmonary congestion and bilateral pleural effusions. TTE revealed severe aortic regurgitation (AR), a 1.7 cm AV vegetation, a small vegetation on the noncoronary aortic leaflet, elevated end diastolic right ventricular pressure, and preserved left ventricular function.

Healthcare providers obtained blood cultures after a single dose of ceftriaxone and before daptomycin was initiated; the cultures remained negative after 5 days. Clinicians became concerned for possible bartonellosis because of the patient’s recent homelessness and negative blood cultures. Clinicians changed the patient’s antimicrobials to doxycycline and rifampin after serologic testing revealed *B. henselae* titers of IgG >1:2,560 and *B. quintana* titers of IgG >1:1,280; IgM titers were negative.

The patient’s renal function continued to decline, likely secondary to glomerulonephritis, and clinicians initiated intermittent dialysis. The patient subsequently refused treatment and laboratory testing but was legally deemed unable to make medical decisions; a court ordered dialysis and cardiothoracic surgery. He underwent bioprosthetic AV replacement and a left atrial appendage ligation on day 29 of hospitalization. The postoperative course was complicated by deterioration of cardiac function and renal failure.

The Wadsworth Center conducted molecular testing of the explanted AV and identified *B. quintana* by using real-time PCR targeting of the *gltA* gene and confirmed the *B. quintana* infection by 16S full gene DNA sequencing of the PCR product. During the hospitalization, the patient completed 10-week courses of doxycycline and rifampin; 6 additional weeks of doxycycline were recommended after hospital discharge. The patient was discharged to a shelter 79 days after admission with hemodialysis scheduled 3 times/week, but he left the facility and did not attend outpatient clinic appointments. He returned to the emergency department 3 months later with volume overload and possible worsening of heart failure (HF), although renal function had recovered. He left against medical advice and was lost to follow-up.

### Case 3 Description

An elderly woman with unknown medical history was found disoriented and lying on a subway platform during the winter of 2020. Bed bugs were noted by emergency responders. She was afebrile, bradycardic, and hypotensive, and it was noted she had hyperpigmented lower extremities with 3+ pitting edema (Table). At the hospital, laboratory values revealed moderate leukocytosis, profound anemia, thrombocytopenia, acute kidney injury, elevated pro-B-type natriuretic peptide (pro-BNP), lactic acidosis, and coagulopathy. TTE showed severe AR with large vegetations on the AV noncoronary and right coronary cusps, moderate pulmonary hypertension, moderate tricuspid and severe pulmonic regurgitation, and diastolic dysfunction. Brain magnetic resonance imaging revealed possible embolic-type infarcts in bilateral cerebral and left cerebellar hemispheres.

Healthcare providers collected blood cultures and then administered ceftriaxone and vancomycin empirically for bacterial endocarditis. Providers started the patient on doxycycline and rifampin 3 days later because the negative blood cultures, homelessness, and the ectoparasitic infestation were suggestive for bartonellosis.

The patient underwent urgent tissue AV replacement and tricuspid valve repair. Warthin-Starry stain was negative. Bacterial cultures held 5 days and fungal cultures held 28 days of the explanted valve were negative. Blood cultures were held for 14 days and remained negative.

The titer for *B. quintana* IgG was <1:128 and the titer for *B. henselae* IgG was >1:1,024; IgM titers were negative. Clinicians changed the patient’s antimicrobial drugs to doxycycline and rifampin because of the clinical suspicion of bartonellosis after consideration of the elevated *Bartonella* spp. titers, negative blood cultures, homelessness, and ectoparasitic infestation. The Wadsworth Center conducted molecular testing of the explanted AV and identified *B. quintana*. The patient died from multiorgan failure 3 weeks after surgery.

### Case 4 Description

A middle-aged man with a history of asthma, bipolar disorder, alcohol use disorder, hepatitis C infection, pancytopenia, and experiencing unsheltered homelessness sought care at an emergency department in the winter of 2022 with dyspnea and bilateral lower extremity edema over the previous 2 months. He was hospitalized for fluid overload with suspected acute HF. At admission, the patient’s vital signs were unremarkable, and no signs or symptoms of systemic infection were noted (Table). Laboratory tests revealed anemia, hypoalbuminemia, and elevated pro-BNP. Chest radiograph showed pulmonary congestion and bilateral pleural effusions.

TTE showed AR, mitral regurgitation, tricuspid regurgitation, mild pulmonary hypertension, and suspected mobile vegetations on the AV. Healthcare providers collected blood cultures before initiating treatment with intravenous vancomycin and ceftriaxone for suspected bacterial endocarditis. Initial and repeated blood cultures remained negative after 21 days. Because of the patient’s recent unsheltered homelessness and reported exposure to cats, dogs, and lice, *Bartonella* endocarditis was considered. Clinicians changed the antimicrobial treatment to doxycycline and rifampin. Serologic testing detected elevated IgG titers to *B. henselae* of 1:2,560 and *B. quintana* of 1:640. *B. henselae* IgM titers were negative.

The patient underwent tissue AV replacement 10 days after admission. Bacterial, fungal, and mycobacterial cultures of the AV tissue were negative. Healthcare providers identified *B. quintana* from the explanted AV by using Sanger sequencing of amplicons obtained from PCR assays targeting 16S rRNA and *ribC* genes. The patient was discharged from the hospital and recommended to complete a 12-week course of doxycycline and 6-week course of rifampin. However, he continued to experience unsheltered homelessness and reported that he was unable to complete the recommended regimen because his medications were stolen.

### Case 5 Description

An ≈60-year-old man experiencing unsheltered homelessness was admitted to an emergency department in summer 2023 after being found collapsed on a sidewalk. Physical examination revealed left-sided facial droop and weakness. He was afebrile, and vital signs were unremarkable. Laboratory testing showed severe anemia and normal renal function (Table). CT scans of the patient’s head revealed an acute right middle cerebral artery infarct with suspected thrombus. CT angiography showed occlusion of the right M1 segment, which was treated with thrombectomy.

Transesophageal echocardiogram revealed mobile vegetations on each AV leaflet, anterior and posterior mitral valve leaflets, and the tricuspid valve; mild AR; and mild to moderate mitral regurgitation. Digital subtraction angiography revealed a 3 mm distal middle cerebral artery mycotic aneurysm.

Healthcare providers collected blood cultures and initiated vancomycin and ceftriaxone for suspected bacterial endocarditis. Blood cultures were held for 5 days and remained negative.

Clinicians considered the patient at risk for *B. quintana* endocarditis because he experienced recent homelessness. They added doxycycline and rifampin after *B. hensalae* and *B. quintana* IgG titers were both 1:2,560; IgM titers were negative. Clinicians discontinued vancomycin and ceftriaxone when cell-free B. *quintana* DNA was detected in the patient’s plasma by using next-generation sequencing.

The patient completed 6 weeks of rifampin and 7 weeks of doxycycline during hospitalization. He did not undergo valve replacement because of stable vegetations and high risk for aneurysm hemorrhage if anticoagulated. He was discharged wheelchair-bound to a shelter. After falling from his wheelchair the next day, he was hospitalized and completed his doxycycline course during an extended rehabilitation hospitalization.

## Discussion

We describe 5 cases of left-sided *B. quintana* endocarditis that occurred during 2020–2023 in persons experiencing unsheltered homelessness in NYC. Most patients had serious complications, including 4 with HF, 3 with renal failure, and 2 with embolic strokes. Renal replacement therapy during hospitalization was required for 2 cases, and 2 patients died.

Echocardiographic findings of AV vegetations prompted evaluations for infective endocarditis. In all cases, negative blood cultures triggered testing for *Bartonella* spp. because clinicians recognized that *B. quintana* infection was a possibility because of the patients’ history of experiencing homelessness. Body lice and homelessness have been linked to *B. quintana* infection (*3*,*9*–*12*,*17*,*19*). In this article, body louse exposure was documented in 3 of 5 endocarditis cases; a fourth patient had what appeared to be bed bugs, which might have been misidentified body lice.

Because of nonspecific clinical manifestations, *B. quintana* endocarditis requires a high index of clinical suspicion to diagnose. Even when clinicians consider bartonellosis, diagnosis can be elusive. The bacterium stains poorly with Gram stain and is visualized better with Giemsa or Warthin-Starry silver stain, although histopathological diagnosis is insensitive and nonspecific (*5*). Diagnosis is commonly made by using serology but can be challenging. Cross-reactivity between different *Bartonella* spp. can affect interpretation (*1*,*16*,*25*–*27*). In the cases reported in this article, titers for *B. henselae* IgG were equal to or higher than for *B. quintana* IgG, and in 1 endocarditis case, *B. quintana* IgG was negative. *B. henselae* and *B. quintana* IgM titers were also negative in all cases, which is expected with prolonged chronic infections.

A standard approach to *B. quintana* diagnosis is lacking. As fastidious organisms, *Bartonella* spp. are difficult to grow by using routine blood culture methods and require up to 3–4 weeks of incubation. *Bartonella* spp. endocarditis blood culture sensitivity can be <20% (*1*,*28*). Periodic subculturing of blood culture broth in shell vials or to solid media with different base formulas has been used, but most clinical laboratories are unable to use these specialized techniques (*1*,*4*,*29*). None of the *B. quintana* endocarditis cases in this article were detected by blood culture and in 4/5 cases cultures were only held for 5 days.

Molecular assays, such as PCR amplification of specific gene targets (e.g., the *Bartonella* citrate synthase gene, *gltA*, *ribC*) and 16S rRNA sequencing of PCR products, are the most reliable methods to detect *Bartonella* infection and can be used for blood or fresh tissue specimens (*4*,*27*,*30*,*31*). In addition, molecular assays can be performed on formalin-fixed paraffin-embedded tissues for retrospective analysis. The diagnostic sensitivity of PCR to detect *B. quintana* from cardiac valve tissue in 1 reference center was 81%, compared with 28% for blood to shell vial culture, 44% for valve tissue to shell vial culture, 33% for blood, and 36% for serum (*4*,*5*). However, molecular diagnosis of *Bartonella* spp. is available in only a limited number of public health and commercial laboratories and tissue is not always available for testing.

In this investigation, molecular testing of excised valve tissue confirmed the detection of *B. quintana* endocarditis in 4 patients, including in 1 patient whose PCR blood test was negative and another with undetectable *B. quintana* IgG. Detection of cell-free B. *quintana* DNA in plasma confirmed the diagnosis in the patient who did not undergo valve replacement.

*B. quintana* infection results in intraerythrocytic and endothelial propagation with biofilm formation that protects it from the immune response and antimicrobial drugs, which might explain the insidious consequences of prolonged bacteremia, resistance to many antibiotic classes, and propensity for recurrent infection (*1*). Experimental and clinical evidence suggest that aminoglycosides have bactericidal activity against *Bartonella* spp. and should be used for >14 days, ideally in combination with a second antimicrobial drug (*32*). Doxycycline plus rifampin has been recommended as an effective alternative regimen (*33*). For *B. quintana* endocarditis, 4–6 weeks of combination therapy is recommended (*1*,*33*,*34*), and valve replacement is often necessary.

A low threshold for diagnostic testing and administration of empiric antimicrobial drugs is necessary when *B. quintana* infection is suspected. Because infections can be asymptomatic and prolonged, the diagnosis and treatment of bartonellosis can be challenging, the consequences of untreated infection can be severe, untreated infection poses a risk of subsequent transmission, and the cost for each prolonged hospitalization (mean = 45 days in this article) is great. Persons experiencing homelessness who are unable to maintain regular clothing hygiene are at an increased risk for body louse infestation and *B. quintana* infection. When evaluating patients with vasoproliferative skin lesions, HF, or cardiac valve vegetations, clinicians should routinely ask about housing status and body lice exposure. In this investigation, clinicians started *B. quintana*–directed antimicrobial drugs for 3 patients within 4 days of their presumptive bacterial endocarditis detection. Those clinicians were in a hospital that frequently treats persons experiencing homelessness.

A history of alcohol use disorder was discovered in 3 of 4 cases with known medical histories, which has been documented previously in patients with *B. quintana* endocarditis (*5*,*14*,*16*,*20*). Those patients also had mental health conditions. Those related conditions may have contributed to delayed detection and poor outcomes.

The burden of *B. quintana* infection in persons experiencing homelessness is unknown. However, we infer from the published literature that this burden might be considerable. In 1999, 25% of clinic patients experiencing homelessness in Seattle, Washington, USA, had elevated antibody titers to *B. quintana*, compared with 1% of blood donor controls (*16*). During 2016–2021, medical centers in Denver, Colorado, USA, reported 12 (85.7%) of 14 patients with *B. quintana* infections were experiencing homelessness (*20*). Over 1-year in Marseille, France, *B. quintana* bacteremia was detected in 10 (14%) of 71 of all patients experiencing homelessness (*3*). With an estimated 653,100 persons experiencing homelessness in the United States (*35*), the potential burden is considerable.

Bartonellosis is not a nationally notifiable disease and is not reportable to the NYC Department of Health and Mental Hygiene. Persons at risk for *B. quintana* infection are also frequently outside the reach of healthcare. Moreover, even if a patient is suspected to have bartonellosis, diagnostic testing is often inconclusive or negative. Therefore, it is likely those cases represent a fraction of all *B. quintana* infections in unsheltered NYC residents during that period.

In conclusion, clinicians who provide clinical services to persons experiencing homelessness should consider *B. quintana* infection when patients have symptoms that may be consistent with bartonellosis, particularly in the setting of prior or current body louse infestation. Available diagnostic testing includes blood and tissue cultures, serologic testing, and molecular assays, which includes cell-free DNA testing; however, negative results do not entirely rule out *B. quintana* infection, and empiric antimicrobial drug treatment should be considered if clinical suspicion for *B. quintana* infection is high.
